# The Typicality Ranking Task: A New Method to Derive Typicality Judgments from Children

**DOI:** 10.1371/journal.pone.0157936

**Published:** 2016-06-20

**Authors:** Farah Mutiasari Djalal, Eef Ameel, Gert Storms

**Affiliations:** Department of Experimental Psychology, University of Leuven, Leuven, Belgium; Center for BrainHealth, University of Texas at Dallas, UNITED STATES

## Abstract

An alternative method for deriving typicality judgments, applicable in young children that are not familiar with numerical values yet, is introduced, allowing researchers to study gradedness at younger ages in concept development. Contrary to the long tradition of using rating-based procedures to derive typicality judgments, we propose a method that is based on typicality ranking rather than rating, in which items are gradually sorted according to their typicality, and that requires a minimum of linguistic knowledge. The validity of the method is investigated and the method is compared to the traditional typicality rating measurement in a large empirical study with eight different semantic concepts. The results show that the typicality ranking task can be used to assess children’s category knowledge and to evaluate how this knowledge evolves over time. Contrary to earlier held assumptions in studies on typicality in young children, our results also show that preference is not so much a confounding variable to be avoided, but that both variables are often significantly correlated in older children and even in adults.

## Introduction

Since the seventies, many researchers studying natural language concepts showed that category membership is graded and that there is a stable within-category structure, usually described as the typicality gradient [1], [2]. This typicality gradient implies that members of a category vary in how good an example they are or how typical they are of the category. For example, for the category of birds, a pigeon is a very good or typical member, in the sense that it is an example that you easily think of when you think of the category, while penguin is a very bad or atypical member, in the sense of it being an example that is rather unusual to think of when you think of the category. Likewise, a chair is a more typical example of the category of furniture than a carpet.

Typicality has been universally acknowledged to be a very important notion in the study of natural language concepts and therefore accurate measurement of this variable is crucial. As Murphy ([3], p. 22) wrote: “Typicality differences are probably the strongest and most reliable effects in the categorization literature”, or as Heit and Barsalou ([4], p. 415) stated “Typicality is arguably a better overall predictor of category performance than any other variable”. Furthermore, typicality has been empirically shown to affect a wide range of variables related to natural language categories, including consistency of category endorsement [5], ease of judging category membership [1], categorization response times [6], [7], exemplar generation frequency [8], speed with which category exemplars are learned [9], usefulness for making inferences [10], [11], priming effects [12], semantic substitutability [13], memory interference effects [14], and so on. As such, typicality offers us probably the best window into the mental representation of semantic categories.

It has been shown extensively that subjects can judge typicality in a very reliable way for most natural language concepts [12], [15–17] and several methods have been proposed to measure typicality judgment. The most widely used method is the typicality rating task which requires the participants to judge the degree of typicality of an exemplar in a particular category on a numeric rating scale. In most research, a six- or seven-point rating scale is used, ranging from 1 for very poor examples of the category to 6 or 7 for very good examples. However, this method can only be used reliably to derive typicality judgments from a population that can appropriately handle numerical values. Therefore, the method cannot be used with very young children who cannot identify the relative values of numbers between 1 and 7 [18], [19]. This level of numerical knowledge has not been achieved yet by 5-year-old children, which forms a serious problem in the study of concept development, where this age is of particular importance [20], [21].

A study from Bjorklund, Thomson, and Ornstein [18] tried to overcome this problem by introducing a method to derive typicality judgment from children using schematic faces. They reduced the 7-point rating scale to a kind of 3-point rating scale by means of three schematic faces, a smiling face, a neutral face, and a frowning face, to indicate the typicality judgments of a very good example an “okay” category member and a very poor example of a category, respectively. They also stressed that the children needed to determine how good an example each item was for the studied category, not how much they liked the items. They compared the results of the children’s typicality judgment derived from the schematic faces with adults’ judgment on a 3-point rating-scale and found that the typicality judgment of the three children groups were highly correlated with the adult ratings. Furthermore, the correlations tended to increase with age.

Several years later, Maridaki-Kassotaki [19] hypothesized that the words “good” and “bad”, used by Bjorklund et al. [18] in the instructions, could lead to a misinterpretation in terms of personal preference rather than typicality. Bjorklund et al. had already tested this possibility and failed to find a significant correlation between typicality ratings and personal preference scores in a different group of children of the same age, but in Maridaki-Kassotaki’s replication using the same group of children for both tasks, she did find a significant correlation. Interpreting this correlation as evidence for a confounding effect, she concluded that the three-schematic-faces procedure of Bjorklund et al. was not a reliable method for deriving typicality judgments from children. Maridaki-Kassotaki proposed an alternative method to derive typicality from children based on family resemblance, a procedure introduced by Rosch and Mervis [22] to predict prototypicality in semantic concepts. Family resemblance was defined as the amount of features an item has in common with other items of that same category. In order to derive family resemblances scores, Maridaki-Kassotaki [19] asked children and adults to generate as many features as possible for the items in every category. For the 5-year-old children, a low correlation was found between family resemblance scores and typicality ratings derived from the three-schematic-faces procedure of Bjorklund et al. [18], but a high correlation was found between children’s family resemblance scores and both adult’s family resemblance scores and typicality ratings. Furthermore, the family resemblance scores from 5-year-olds did not significantly correlate with their personal preferences. From these results, Maridaki-Kassotaki concluded that family resemblance scores yield a reliable method to derive typicality judgments, not only from adults, but also from young children.

However, there are some methodological problems concerning the research of Maridaki-Kassotaki [19]. First of all, she compared several typicality scores of children with those of adults using the adults’ scores as the standard. Yet, previous research [18], [20], [23] has found that the mental representations of categories are not robust over time, but gradually converge to adult mental representations. In order to further examine this, typicality data from different age groups, starting from the earliest age where typicality judgments can be meaningfully gathered, should be compared.

Second, based on a high correlation in 5-year-olds between typicality ratings derived from the three-schematic-faces procedure of Bjorklund et al. [18] and personal preference data, Maridaki-Kassotaki stated that the method of Bjorklund et al. was not suitable to derive typicality judgments from children. However, she never gave any rationale why there should not be any relationship between typicality and preference and this has never been thoroughly explored so far in adults, nor in older children. It is possible that typicality is related to personal preference, even in adults. Furthermore, Maridaki-Kassotaki measured the correlation between typicality and preference group-wise, that is, across participants, while personal preference can differ widely between persons. To draw a conclusion about the relation between typicality and personal preference, this correlation should be calculated on an individual level and in different ages.

Third, the family resemblance measure assumes a particular concept representation, where exemplars that resemble the central tendency of a category more will be judged more typical. Several studies, however, challenged this central tendency idea and presented representation models that start from ideals, rather than from averages [15], [24], [25].

The main purpose of the present study is to evaluate an alternative method that allows researchers to measure typicality judgments in young children who cannot handle numerical information yet. The proposed method, which could push the age limit to study typicality from 9-year-olds down to 5-year-olds, uses a ranking rather than a numerical rating procedure, making use of the well-established finding that young children, even pre-verbal infants, are able to reliably perform ordinal judgments (e.g., [26]). An additional advantage of ranking over rating is that in adults, agreement among participants in typicality ranking is greater than in rating [27]. The procedure further relies only to a limited extent on linguistic knowledge in the sense that participants are expected to understand the task instructions, but the use of the words “typical” and “atypical” is avoided. Finally, the method aims to be a theory-free measurement instrument that is compatible both with a central tendency view on concept representation and with an ideal-based representation.

The usefulness of the alternative method will be measured based on three requirements. First, since the rating method is generally considered a reliable method to measure typicality, the reliability of the ranking data obtained from an age group should be equally high as that of rating data from the same age group and the reliability of the ranking data of the youngest children (who are not yet able to do the rating task) should not be much lower than that of older age groups. This would prove that the ranking method is as reliable to measure typicality as the standard rating method. Second, if the ranking task is a valid alternative method to derive typicality judgments, the ranking results should correlate strongly with the results of the standard rating procedures. Third, since ratings obtained from different ages converge slowly towards the typicality ratings of adults as the children get older, a similar converging pattern should emerge in the rankings. This would be evidence that the ranking method is able to depict the evolvement of typicality as in the rating method.

An additional goal of the paper is to investigate the possibly confounding influence of preference on typicality by studying the relation between typicality judgments and personal preferences both in children and adults on an individual level.

Three studies were performed. In the first study, adults’ typicality ranking data were compared to adult standard 7-point-scale typicality rating data, since the latter has proven to be a valid method to derive typicality ratings from adults [22], [28], [29]. In a second study, similar tasks were conducted and compared in children. More specifically, 9- to 14-year-olds were asked to perform a 7-point-rating scale task. A typicality ranking task was presented to participants of the same age groups, but also to younger children aged 5 and 7 and the results of the two procedures are compared. Finally, in a third study, the relation between typicality and preference is investigated systematically in the different age groups by correlating these measures on an individual level, since personal preference is a subjective measurement that is not necessarily robust across participants.

## Study 1: Adults’ Typicality Rating and Ranking

In the first study, an alternative method of typicality measurement was introduced to the adults. A method that is based on typicality ranking was proposed. In this method, items are gradually sorted according to their typicality. As a comparison, typicality ratings were also gathered from a different group of adults using a 7-point-rating scale. The results of the typicality ranking task were compared with the typicality ratings in order to test whether the ranking method is a reliable alternative to derive typicality judgments. We predicted that scores derived from the ranking task would significantly correlate with scores gathered through a standard typicality rating task.

### Methods

#### Ethics statement

Study 1 was conducted with the approval of the Social and Societal Ethics Committee (SMEC) of the University of Leuven. Written informed consent was obtained from all participants before starting the task.

#### Participants

A total of 42 adults (average age 29 years 1 month; *SD* = .40) performed the typicality ranking task. The adults were friends and colleagues of the researchers, recruited in Flanders, Belgium. Each of the participants performed the task for only half of the categories, thus four out of eight categories. Concretely, half of the participants performed the tasks for the categories *birds*, *kitchen utensils*, *vegetables*, and *vehicles* (group 1), the other half for the categories *fruit*, *mammals*, *musical instruments*, and *tools* (group 2). Participants were randomly assigned to one of the two groups. In order to examine the relation between preference and typicality on an individual level, this group of participants also performed the preference task that will be described in Study 3.

Twenty-four subsequent adult participants (average age 24 years and 1 months; *SD* = 3.69) performed the standard typicality rating task on a 7-point scale.

#### Materials

The stimuli were 96 colored pictures printed on 10 x 15cm cardboard representing each exemplar from eight categories. There were four artifact categories (*kitchen utensils*, *musical instruments*, *tools*, and *vehicles*) and four of natural categories (*birds*, *fruit*, *mammals*, and *vegetables*). Each category contains 12 of possible exemplars. The exemplars were selected from a norm study of De Deyne et al. [16]. The selection criteria were adult typicality and estimated age of acquisition, two variables which were collected by De Deyne et al. [16]. Initially, the minimum age of acquisition was set at five years old, since this was the age of our youngest children. However, this age criterion resulted in a too small set of items for certain categories. For example, for the category of *birds*, only two item names were estimated to be acquired before the age of five (chicken and duck). Therefore, we decided to increase the minimum age of acquisition to seven years old in order to be able to select at least 12 exemplars for each category. Besides age of acquisition, items were also selected on the basis of their adult typicality score in order to make sure that the selected items were representative for the full range of typicality within a category. Based on the adult typicality ratings, we selected for each category 4 typical examples, 4 atypical examples and 4 moderately typical examples. The 12 items selected for each category are presented in the S1 Appendix. The category of *insects* was used to illustrate the idea of typicality. This example category contained two typical items (mosquito and fly) and two atypical items (butterfly and worm). As with the target categories, these items were chosen on the basis of age of acquisition (learned before the age of seven) and adult typicality. A pilot study with two 5-year-olds revealed that these children were familiar with all the items of the eight categories and the example category, except for one, even though the estimated age of acquisition of the names of many more items exceeded the age of the pilot participants. The item “asparagus” was not recognized. Nonetheless, we decided to include this item in the final stimulus set. This decision was taken after we calculated, for each age group and for both typicality ranking and rating tasks, Spearman-Brown reliability estimates and Pearson correlation without the item “asparagus” and compared it with the actual data set (in which the item “asparagus” was included). The results show the differences are not significant.

#### Procedure

All the participants were tested in a quiet room. The participant and the experimenter sat at a table facing each other. Before the actual task started, the experimenter explained the instructions and the participant was given some examples to practice. The examples were four exemplars from category *insects* (mosquito, fly, butterfly, and worm) and the experimenter explained that some examples—like mosquito and fly- are good examples of the category *insects*, while other examples—like butterfly and worm- are poor examples. Once it was clear that the participants understood the concept of typicality, the actual typicality ranking task was conducted. All the 12 items from a certain category were spread out on the table so that the pictures were visible. As with the practice category, participants were first told the category name and asked to look through the items to indicate whether they knew all items’ names or were familiar with the objects presented. If not, participants were told the name of the objects. Next, participants were told that all 12 items belonged to the mentioned category and that items might differ in how good an example they are for that particular category. Participants were told that an item can be a good example of the category or rather a bad example.

The actual ranking task consisted of three stages which each participant had to go through. In the first stage, the participants were asked to construct two piles: one pile with the items that she considered to be good examples of the category, further called the “good example pile” and another pile with items that she considered to be bad examples, called “bad example pile”. Participants were explicitly told to judge how good the items are as examples for the particular category and not to base their judgment on how much they liked the items. They were also told that the two piles could differ in size. After this first stage, the bad example pile was taken away and participants were asked to look at the items of the good example pile and to construct again two piles: one pile with very good examples of the category (the “best example pile”) and one pile with somewhat less good exemplars (the “less good pile”). Participants were subsequently asked to repeat this procedure with the bad example pile, which resulted in a “less bad example pile” and a “worst example pile”. Finally, participants were asked to order the items of each of the four piles according to their *goodness* starting with the item they thought of as the best example and ending with the one they considered as the worst example. Participants were instructed to start with the best example pile and to rank these according to their *goodness*. The items of the less good pile, the less bad pile and the worst example pile, were successively added to the ranking, one pile at a time. This procedure was repeated four times, for four different categories.

The three-stage procedure simplified the ranking procedure, since subjects initially had to sort all items into two and subsequently into four piles before making a typicality ranking of all the items.

For the standard typicality rating task, all 12 items of a category were put randomly in front of the participants. Once they had looked through the pictures, participants were asked if they were able to name all items presented and if they were familiar with the objects. Next, the experimenter explained that all items belonged to the same category and that they might be different in how good an example they are of the particular category. Contrary to the typicality ranking task, all items were taken away and presented again to the participant one at a time. Participants were now asked to rate each item on a 7-point rating scale according to their typicality with 1 being a very bad example of the category and 7 being a very good example of the category. This procedure was repeated for each of the eight categories. As with the typicality ranking task, the instructions were first made clear with the example category of *insects*.

For both tasks, the order of category presentation as well as the order of item presentation was counterbalanced across participants.

### Results and Discussion

In the typicality-ranking task, each item received a score ranging from 1 to 12 according to its position in the typicality ranking of a participant. Score 1 indicates the worst example of the category and 12 indicates the best example in the category. The final typicality ranking score for an item, then, is the average of scores across participants. The higher any of the typicality scores, the higher the perceived typicality of the item was.

For the typicality rating task, the items were given a score between 1, indicating the worst example, and 7, indicating the best example. Scores for each item were then averaged across participants. A high averaged typicality rating score reflected the best example, while the worst example of a category was reflected by a low averaged typicality rating score.

The consistency of the typicality rankings and ratings over the participants was computed using the split-half method followed with the Spearman-Brown formula (see [30]). This was done by splitting the number of participants randomly into two parallel halves (r_half_). Then the correlation was computed between averaged typicality scores of these two half splits and replicated for ten thousand random splits. The halves-reliability estimates were then adjusted using the Spearman-Brown prediction formula (2*r_half_ /(1+ r_half_)) and averaged across the distributions. Table 1 shows these estimated reliabilities for the typicality ranking and typicality rating scores for the eight categories. As can be seen, all reliabilities exceeded .99. These results indicate a very high consistency between subjects, with virtually no difference between the two tasks.

**Table 1:** Reliability estimates and Pearson correlations between the typicality rating and ranking scores for the eight categories.

Category	Reliability		
	Typicality ranking	Standard typicality rating	Pearson correlation
Birds	.97	.98	.92*
Fruit	.99	.99	.96*
Kitchen utensils	.97	.97	.98*
Mammals	.98	.97	.94*
Musical instruments	.99	.98	.97*
Tools	.98	.98	.96*
Vegetables	.97	.97	.94*
Vehicles	.99	.98	.97*

*correlation is significant at the .0001 level

To evaluate whether the typicality rating is a valid method to derive typicality scores, Pearson correlations were computed between the typicality rating scores and the alternative method of typicality ranking scores. The results, as can be seen in Table 1, revealed significant (*p* < .0001), very high correlations for all studied concepts.

In sum, the typicality ranking task shows equally high between-subject consistency as the standard typicality rating task. The high correlations between ranking and rating scores strongly suggest that the newly introduced typicality ranking task is a valid method to derive typicality judgments from adults. We expected similar results for the children as well. Therefore, in Study 2, the same two methods were used to collect typicality judgments in groups of children with different ages to see whether ranking is also a valid method to derive typicality from children.

## Study 2: Children’s Typicality Rating and Ranking

Even though the typicality ranking task was proven to be a valid method to derive typicality from adults in Study 1, the question still remains whether the task is also appropriate to measure typicality in children, especially in children that are too young to master numerical information. Therefore in Study 2, children’s typicality judgements, gathered in a typicality rating task, were compared, within the same age group, with typicality judgements from the ranking task.

### Methods

#### Ethics statement

Study 2 was conducted with the approval of the Social and Societal Ethics Committee (SMEC) of the University of Leuven. Written informed consent was obtained from all the adult participants and the parents (on behalf of the children who enrolled in this study) before starting the task.

#### Participants

A total of 210 children, from five different age groups, performed the typicality ranking task: 42 5-year-olds (average age 5 years and 6 months; *SD* = .27), 42 7-year-olds (average age 7 years and 8 months; *SD* = .29), 42 9-year-olds (average age 9 years and 9 months; *SD* = .30), 42 11-year-olds (average age 11 years and 8 month; *SD* = .31) and 42 14-year-olds (average age 14 years and 2 months; *SD* = .20). The children were recruited from Dutch-speaking kindergartens and elementary and high schools in Flanders, Belgium. The native language of all participants was Flemish (Belgian Dutch). The 5-, 7- and 9-year olds had very limited knowledge of French or English, and the 11- and 14-year olds had minimal additional knowledge of French and English through formal instruction at school that started at the age of 11. All of the schools were public institutions that have pupils populations that are representative of the Belgian population in terms of socio-economic status and cultural background (which means a vast majority of Caucasians and a small minority of second generation immigrants).

Half of the participants of each age group conducted the typicality ranking task, as well as a personal preference task (to be described in Study 3), for the categories *birds*, *kitchen utensils*, *vegetables* and *vehicles* (group 1), the other half for the categories *fruit*, *mammals*, *musical instruments* and *tools* (group 2). Participants were randomly assigned to one of the two groups.

A different group of children ranging from 9 to 14 years performed the typicality rating task on 7-point-scale. There were in total 44 children from three age groups: 17 9-year-olds (average age 9 years and 8 months; *SD* = .57), 15 11-year-olds (average age 11 years and 9 months; *SD* = .35), and 12 14-year-olds (average age 13 years and 11 months; *SD* = .33). The children were recruited in different schools in Flanders, Belgium. They performed the typicality rating task on a computer for all the eight categories. We did not gather typicality rating data from children of 5 and 7 years old since the procedure requires more linguistic and numerical knowledge than such young children have acquired so far (as is recognized in [18], [19]).

#### Materials

Stimuli were the same eight categories used in Study 1, with four artifact categories (*kitchen utensils*, *musical instruments*, *tools*, and *vehicles*) and four of natural categories (*birds*, *fruit*, *mammals*, and *vegetables*).

#### Procedures

In general, the same procedure was used to conduct the typicality ranking task as in Study 1, except for the youngest group (children aged 5), where the experiment was conducted in a more playful context. The children of 5 years old were asked to imagine a friendly spaceman being lost on earth who had come to their school. The spaceman did not know what X was, with X any of the example category (*insects*) or target categories (e.g., *birds*). To help the spaceman understand what X are, the experimenter told that it would be a good idea to give the spaceman a few examples of X: Some examples are good to give the spaceman an idea of what X are. These are examples that people would quickly think of when they think of X. Others are bad or not suitable to give the spaceman an idea of what X are. These are items that would not quickly cross people’s mind if they think of X. This playful cover story was used both to explain the concept of typicality by means of the example category and to introduce each of the target categories in the actual typicality ranking task. For the older children (7-, 9–11-, and 14-year-olds), the spaceman story was not used in the instruction.

Due to their limited attention span, children of 5, 7, 9 and 11 years old provided typicality ranking data in two separate sessions of half an hour each (two categories per session). The order of category and item presentation was counterbalanced across participants.

For the standard typicality rating task, children performed the task individually on a computer accompanied by the researcher. All the items were shown on colorful pictures presented one by one on the screen and the participants were asked to click a number that reflected their typicality judgments. Before proceeding to the actual task, the participants were given six trial questions where they had to rate typicality judgments on each of the six examples from the category *clothes* (pants, socks, skirt, t-shirt, shoes, and hat). They were asked to rate each item on a 7-point rating scale with 1 indicating a very bad example of the category and 7 indicating a very good example of the category. Once they understood the instruction, the actual was conducted. The participants gave their typicality judgments for all the 96 items.

### Results and Discussion

Typicality ranking scores were calculated in the same way as in Study 1. Each item received a score from 1 to 12 according to its order in the ranking made by every participant. For the typicality rating scores, each item received a score ranging from 1 to 7 from every participant. Both for typicality ranking and the typicality rating, the scores were summed across participants for each age group and for each item. The higher the summed typicality score, the higher the perceived typicality.

#### Reliability of typicality ranking and rating scores

The consistency of the typicality scores in an age group was computed using the same method as in Study 1, (i.e., split-half method combined with the Spearman-Brown formula) for both tasks separately. For the typicality ranking task, most reliability estimates were higher than .85. For only one category for 7-year-olds reliability dropped below the threshold of .85 (i.e., *mammals*: .80), and half of the studied categories for the youngest age group yielded only modest reliabilities: *musical instruments* (.59), *vehicles* (.71), *tools* (.48) and *mammals* (.65). Even the 5-year-olds, however, showed reliability coefficients up to .91 for the category of *birds*. These results suggest that the limit for using the ranking method will be around the age of 5, as the internal consistency is high for half of the categories, but drops to moderate for the other half. For the typicality rating task, the reliability estimates were higher than .80 for all tested age groups and all categories.

An ANOVA was performed on the Spearman-Brown coefficients for typicality ranking scores with age and category as independent variables. We found that only the effect of age was significant, *F*(5, 35) = 12.64, *p* < .0001. In order to see whether there is a significant increase or decrease in reliability estimates with age across all eight categories, an analysis using contrast matrices with age as a fixed effect was run. The results revealed that there is a significant positive effect of age (*t* = 4.728, *p* < .0001), where the reported t-statistic was treated as z-statistic to derive p-value. This result suggests that reliability estimates tend to increase across age.

In order to have a clear overview of the comparison between the two tasks, the reliability estimates were averaged across all the eight categories. First, each of the reliability estimates from both tasks was transformed into Fisher *Z’* scores, then these scores were averaged across categories. The averaged *Z’* scores were then re-transformed back into Spearman-Brown coefficients. The results for the typicality ranking task from 5-, 7-, 9-, 11-, 14-year-olds, and adults were, respectively, .79, .92, .95, .96, .96, and .98; and for the typicality rating task from 9-, 11-, 14-year-olds, and adults were .92, .95, .95, and .98, respectively (see Table 2). The results showed that both of the tasks, typicality ranking and rating, are reliable methods to derive typicality for children in the sense of being inter-individually consistent in the tested age groups. More specifically, the reliability estimates of the typicality ranking were equally or more stable than the ratings from 9-year-olds onwards. They drop slightly in the 7-year-olds (.92), and decrease further in the 5-year-olds to the still acceptable level of .79. Note further that in the typicality rating task, the consistency dropped slightly sooner, from 11- (.95) to 9-year-olds (.92). In sum, the new alternative method of gathering typicality using the ranking task yields good typicality from young children.

**Table 2:** Pearson correlations and corrected correlations between typicality ranking and rating.

Age groups	Reliability		Pearson	Adjusted
	rating	ranking	correlation	correlation
5 y/o	-	.79	-	-
7 y/o	-	.92	-	-
9 y/o	.92	.95	.92**	.98**
11 y/o	.95	.96	.89*	.93**
14 y/o	.95	.96	.85*	.89*
adults	.98	.98	.96**	.98**

*correlation is significant at the .001 level

**correlation is significant at the .0001 level

#### Correlations between typicality ranking and ranking

To test whether typicality ranking can be used as a method to derive typicality scores from children, one does not only need to show that the rankings are reliable, but also that they provide a valid measure. Therefore, Pearson correlations were computed between the typicality ranking scores and typicality rating scores from children of 9-, 11-, and 14-years-old. First, the correlations were computed for all the eight categories. The results revealed significant (*p* < .01) correlations, with most of the values exceeding .75, except for the category *tools* for 11-year-olds (.69) and *vegetables* for 14-year-olds (.38). Next, these correlations were transformed into Fisher’s Z’ scores and then averaged across categories. These averaged *Z* scores were re-transformed back into correlations. In order to see what the correlation would be if one could measure the typicality ranking and rating with perfect reliability, the adjusted correlation (i.e., after *correction for attenuation*; a method developed by Spearman [31], to avoid possible error variance in a measurement that provides more accurate estimations) was also computed by dividing the empirically obtained correlation between typicality ranking and rating by the product of the square root of the reliability coefficients of the typicality ranking and the typicality rating tasks. The results (see Table 2) showed that all the correlations were high and significant (*p* < .001), even for the 9-year-olds (*r* = .92 and adjusted *r* = .98).

#### Correlations between age groups

Pearson correlations were again computed to explore the evolution of typicality scores across age for both the ranking and the rating task. First, correlations were computed *between* age groups for all the eight categories in each task. These correlations were then transformed into Fisher’s Z’ scores and then averaged across categories. Next, these averaged *Z* scores were re-transformed back into correlations. The results can be seen in Table 3 for the ranking task and Table 4 for the rating task.

**Table 3:** Pearson correlations between age groups for the typicality ranking task.

	5 y/o	7 y/o	9 y/o	11 y/o	14 y/o
7 y/o	.83**	-	-	-	-
9 y/o	.85**	.91***	-	-	-
11 y/o	.77*	.86**	.95***	-	-
14 y/o	.75*	.82*	.93***	.95***	-
Adults	.67*	.72*	.88**	.94***	.94***

*correlation is significant at the .05 level

**correlation is significant at the .001 level

***correlation is significant at the .0001 level

**Table 4:** Pearson correlations between age groups for the typicality rating task.

	9 y/o	11 y/o	14 y/o
11y/o	.93*	-	-
14 y/o	.89*	.92*	-
adults	.88*	.94*	.94*

*correlation is significant at the .001 level

Significant correlations were found between all age groups in both tasks. These correlations indicate that children and adults agree well upon typicality. Furthermore, agreement upon typicality scores increases gradually as the age gap becomes smaller. These gradual increases are clear in the typicality ranking task, where the correlation is higher whenever the age difference of any pair of age groups is smaller, and this was even the case for the young children of 5 and 7years old. For instance, the correlation between 7- and 9-year-olds was higher than the correlation between 7- and 11-year-olds. For the typicality rating task, again the correlations between age groups were all significant, but the gradual increase as the age gap decrease, however, could not be observed always. Furthermore, the correlations for the ratings were also slightly lower than the corresponding values for the typicality ranking task.

#### Typicality in children and adults

The results show that there is a clear evolution in the correlations between age groups in the typicality ranking task, where the correlation is higher whenever the age difference of any pair of age groups is smaller. This finding suggests that children and adults differ in their mental representation of category structure, and as children get older, they gradually attend to the adults’ category structure. For example, the item *potato* in category *vegetables* was endorsed more as typical item by 5-year-olds (*M* = 7.71) than the adults (*M* = 2.38), as well as *sled* in category *vehicles* (*M*_5-year-olds_ = 5.90 and *M*_adults_ = 1.43), or *seagull* in category *birds* was endorsed less by children aged 5 (*M* = 4.76) than the adults (*M* = 9.14).

Summarizing, the results of Study 2 indicate that even from a very young age onwards, the averaged typicality ranking of children and adults is both a reliable measure, as shown in the high consistency over rankings of different participants within every age group, and a valid measure, since the derived scores correlate highly with the generally accepted (golden) standard of ratings. Moreover, the fact that the convergence pattern to the adult typicalities as children grow older is also clearly present in the ranking data provides further evidence for its validity.

## Study 3: Children’s and Adults’ Personal Preferences

In Study 3, the relationship between typicality and personal preference for adults and its evolution across age were explored. Furthermore, as a critic to Maridaki-Kassotaki’s [19] study, in which she correlated typicality and preference data that were aggregated over participants from an age group, we will calculate correlations on an individual level, since personal preference may not be robust across participants. Furthermore, if there is indeed a close relationship between typicality and personal preference in adults, this would be reflected in a significantly high correlation between both scores for adults. For the evolution of the correlation between typicality and preference, there are several possibilities. On the one hand, it is possible that young children, as Maridaki-Kassotaki claimed, misinterpret typicality in terms of preference because of their limited numerical and linguistic knowledge. In that case, we predict that the correlations between typicality and preference decrease with increasing age. On the other hand, if typicality and personal preference are closely related to each other in adults, the correlation between typicality and preference might gradually increase over time to converge to the adult correlation. A third possibility is the lack of evolution over time. If the typicality ranking task does not rely on elaborated numerical and linguistic knowledge, misinterpretations of typicality in terms of preference might be minimal, implying that no evolution of the relation between typicality and preference over time is expected.

### Methods

#### Ethics statement

Study 3 was conducted with the approval of the Social and Societal Ethics Committee (SMEC) of the University of Leuven. Written informed consent was obtained from all the adult participants and the parents (on behalf of the children who enrolled in this study) before starting the task.

#### Participants

The typicality ranking scores for the six age groups, as gathered in Study 1 and 2, were reused in this study. Personal preference scores were collected from the same participants.

#### Material

Stimuli were the same eight categories used in Study 1 and 2.

#### Procedures

All twelve items of a category were put on the table in a random order so that all the pictures were visible. Participants were asked to look at all the items and pick out the item that they preferred most. The most preferred item that was then put aside and again they were asked to pick out the most preferred of the remaining 11 items. The selected item was taken away after every choice. This procedure was repeated until all 12 items were ordered according to the participant’s preference.

Participants provided personal preference data for four different categories. As in Study 1 and 2, category and item presentation was counterbalanced across participants. As mentioned earlier, the same participants in Study 1 (adults) and Study 2 (children) performed both the ranking and the preference tasks. The order of the tasks was fixed: the typicality ranking task was always conducted prior to the personal preference task, in order to eliminate the possible undesirable effect of personal preference on the typicality ranking task [19].

### Results and Discussion

The typicality ranking scores for all categories were taken from the previous studies. The personal preference scores were derived by assigning a score to each item, ranging from 1 to 12 according to the participant’s preference. A score of 1 was given to the least favorable item of the participant; a 12 was given to the most favorable item.

#### Subjectivity in personal preference

In order to demonstrate that preference is a person-specific, subjective measurement, variance of typicality scores and the personal preference scores were compared for each category. First, the variance of each item from a particular category over participants was calculated. Next, these variances were averaged across the 12 items. This was done for both tasks, typicality and personal preference, and for every age group in all the eight categories. The variance of the typicalities and of the personal preferences was then compared. In 41 out of 48 cases (8 categories and 6 age groups) the variance of the preference scores exceeded the variance of typicality scores, showing that there is more variability in the personal preferences scores then in the typicality. This was the case in all age groups, even in the young children.

#### Relationship between typicality and personal preference

To investigate the relation between typicality and personal preference, we calculated for every category the correlation between both measures on an individual level for every participant of each age group. The percentage of participants that show a significant correlation was counted. In all six age groups and in all categories, we found at least 25% of the participants (6 out of 21 participants) showing a significant correlation between typicality and preferences, except for 11-year-olds in category *vehicles* (only 1 participant out of 21), 14-year-olds in category *mammals* (5) and *birds* (3), and adults in category *birds* (5). These findings clearly illustrate that there is a relation between typicality and preference, even in adults.

**Fig 1:**
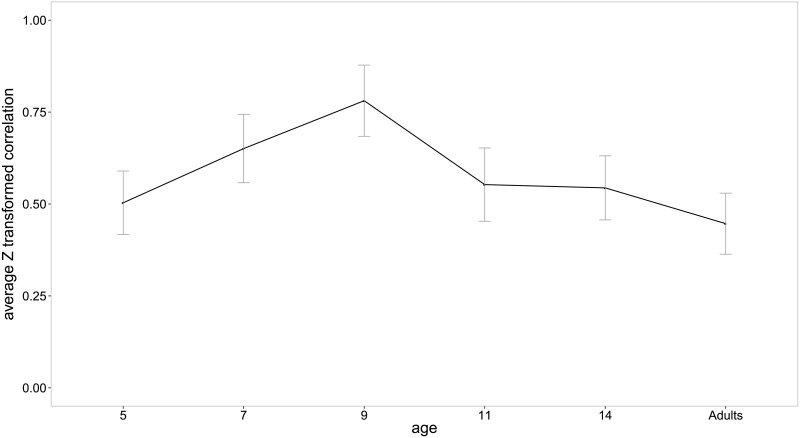
*Z*’-transformed correlation between typicality ranking and preference averaged across category for each age group.

We further studied the evolution of the correlation between typicality and preference across age groups. Fig 1 contains the Z’- transformed correlations averaged across category for every age group. A mixed effects analysis was performed on the Z’- transformed correlations to test the effect of age (5-, 7-, 9-, 11-, 14-year olds, and adults) and the effect of category. A random effect of participants was also included in the analyses. The analysis was carried out in R (version 3.1.2) using the lme4 package [32]. Four models were fitted in order to test the main effects of age and category and the interaction between the two. The first model had two random effects for participants (in which participants are allowed to have a different intercept and can vary in the effect of category) and two fixed effects (age and category). The second and the third models were identical to the first model except that only one fixed effect was included in each; the fixed category effect was eliminated in the second model; and the fixed age effect was eliminated in the third model. The fourth model was identical to the first model, except for the addition of the interaction term (i.e., age*category).

To test the main effect of category, the first and the second model were compared. The results show that the first model provided a significantly better fit to the data (χ^2^(7) = 119.37, *p* < .0001). This means that there were significant differences in the correlation between typicality and preference across categories. The main effect of age was tested by comparing the first model and the third model. The results show that the first model, where the age effect was included, fits the data significantly better (χ^2^(5) = 37.69, *p* < .0001). This result suggests that the relation between typicality and preference differs among the six age groups. Finally, to test the interaction between age and category, the first and the fourth model were compared. The results revealed that the effect of age on the relationship between typicality and preference differs in different categories (χ^2^(35) = 103.05, *p* < .0001). Since the effect of age was significant, an additional analysis was run in order to see whether there is a significant (linear, quadratic, or cubic) trend in the correlation between typicality and preference across age. Analyses using contrast matrices with age as a fixed effect revealed significant negative quadratic trends (see Fig 1). We report the t-statistic and treat it as a z-statistic to derive p-values (*t* = -2.347, *p* = .019). This means that the relationship between the typicality and preference starts to increase from the young children and reaches the peak on 9-year-olds and decreases gradually towards the adults.

Further, we estimated the age effect for each category separately. The same analyses were carried out to test the simple main effect of age, but this time only two models were compared for each category. The first one was similar as the second model described above, where only one main effect of age and two random effects of participants were included. The second model was similar, but without the age effect. The results revealed that in five out of eight categories, the effect of age was significant and quadratic trends were found: *birds* (χ^2^(5) = 29.29, *p* < .0001 and *t* = -3.46, *p* = .0005), *fruit* (χ^2^(5) = 12.51, *p* = .028 and *t* = -2.66, *p* = .008), *musical instruments* (χ^2^(5) = 20.39, *p* = .001 and *t* = 2.67, *p* = .007), *vegetables* (χ^2^(5) = 51.62, *p* < .0001 and *t* = -4.64, *p* < .0001), and *vehicles* (χ^2^(5) = 13.82, *p* = .017 and *t* = 2.05, *p* = .04). In category of *kitchen utensils*, *mammals*, and *tools*, the effect of age was not significant: *p* = .08, *p* = .46, *p* = .16, respectively.

For the categories of *birds*, *fruit*, and *vegetables*, this relation first increased and then decreased over age, while the reversed pattern was found in the categories *musical instruments* and *vehicles*. For the other categories, no clear age trend was present. In sum, a clear relation was found between typicality and personal preference, even in adults. Also in children at different ages, typicality and personal preference were closely related to each other.

## General Discussion

The purpose of the studies described in this paper was to develop an alternative method to derive typicality scores from young children using a typicality ranking instead of typicality rating task. Like the family resemblance based method of Maridaki-Kassotaki [19], our newly developed typicality ranking task does not rely on participants’ numerical knowledge and only to a limited extent on linguistic knowledge. Unlike the family resemblance method, which assumes that categories are represented by an abstract central tendency, the method to derive typicality is independent of any representational theory.

Three studies were conducted in six different age groups (5-, 7-, 9-, 11-, 14-year-olds, and adults) to investigate whether a typicality ranking task is a valid method to derive typicality scores. Four artifact categories (*kitchen utensils*, *musical instruments*, *tools*, and *vehicles*) and four of natural categories (*birds*, *fruit*, *mammals*, and *vegetables*) with 12 possible exemplars in each category were used in all the three studies. In Study 1, the typicality ranking task was introduced to the adults and was compared to the standard 7-point-scale typicality rating task. In Study 2, the same method was conducted to children. Finally, in Study 3 the relation between typicality and personal preference was explored.

### Towards a new method to derive typicality judgments from children

The typicality ranking task was compared to the standard 7-point-scale typicality rating task in order to test whether the new alternative method is a reliable and a valid method to derive typicality judgments. We found that the reliability estimates from the typicality ranking were high and, in line with the finding from Barsalou [27], the rankings were slightly more stable in most age groups in comparison with the typicality rating task. We also found high correlations between ranking and rating scores both in adults (Study 1) and children (Study 2). Further, the correlation *between* age groups were compared and revealed that convergence towards adult typicality increases gradually as the age gap becomes smaller.

The results for the rankings should not come as a surprise. It is a well-established finding in the psychology of mathematics that children master ordering very early in their development, even before they learn to count verbally [26]. Also in terms of measurement theory, the requirements for a participant in the rating task are higher than those in a ranking task. In the former, judgments at an interval level are required, while ordinal information is needed in the ranking task [33].

Our findings show that deriving typicality from averaged rankings in an age group succeeds in lowering the age from which graded structure in semantic categories can be obtained from primary school children to pre-school aged children. However, our results also suggest that the age of 5 probably approaches the limit at which children are capable of judging the goodness of examples of a category, because the inter-person consistency with which the rankings are made decreased from very high for all categories in 7-year-olds to moderate for half of the categories (and high for the other half) in the 5-year-olds. Given the fact that children are able to give order judgments (like quantity, size, etc., of concrete stimuli) at an age considerably younger than 5 years, the decreasing consistency should be attributed to the specific task, that is, judging gradedness in semantic categories, rather than the general cognitive ability of ordinal judgment.

One might wonder whether averaging over the essentially ordinal measures of different subjects is allowed. Strictly speaking, however, rating scale information might also fail to result in trustworthy interval data. The fact that the correlation between the averages of the two different methods is very high can, though, reassure us of the tenability of the assumed numerical properties in the averaging process. Furthermore, the increasing pattern of the correlations as the age gap between groups decreases provides further evidence for this. Finally, the high consistency of the ranking data, also reported in Barsalou [27], argues for treating the average ranking as a reliable measure of typicality in an age group.

### Typicality and personal preference

While Maridaki-Kassotaki [19] claimed that a typicality measure can only be suitable to derive typicality judgments from children if that measure is not related to personal preference data, we found significant correlations between typicality and personal preference, even in adults, when correlations were calculated on an individual level. These results suggest that personal preference might play an essential role in typicality perception (or vice versa). Another possibility is that the relation between typicality judgment and personal preference is mediated through a third factor. This factor might be mere exposure, frequency of exposure or familiarity with a category item. Previous research already showed a significant relation between typicality judgments and familiarity [15], [29], [34]: The more familiar we are with a certain category member, the more typical we think that member is of the category. It is very likely that the more familiar we are with an object the more we like it. In the field of social psychology, research on the phenomenon of mere exposure has provided evidence for the fact that people have a higher affinity for those things they have been repeatedly exposed to [35], [36]. It is not surprising that those items that are most frequently encountered, are also the ones we are most familiar with, and hence, are also perceived as most typical for the category. Yet another factor that could mediate the relation between typicality and preference is actual conceptual knowledge of the studied categories, as shown by [37]. More research is needed to explore the relation between typicality, personal preference, familiarity, frequency of exposure and conceptual development.

## Conclusion

The typicality ranking task is shown to be a good, direct and brief alternative to derive typicality judgments from both adults and children from the age of five years onwards. In the typicality ranking task, items are gradually sorted according to their typicality. Contrary to the typicality rating task, no advanced numerical or linguistic knowledge is required from the participants, which makes the task suitable for young children. Its validity was demonstrated by high consistency between subjects and high correlations with the standard typicality rating task. The results show that the typicality ranking task can be used to assess children’s category knowledge and to evaluate how this knowledge evolves over time.

## Supporting Information

S1 Appendix Selected items for the eight target categories. (DOCX)
