# Supplementary material for: The Typicality Ranking Task: A New Method to Derive Typicality Judgments from Children
Source: PLoS One. 2016 Jun 20;11(6):e0157936. doi: 10.1371/journal.pone.0157936 (PMC4913967; doi:10.1371/journal.pone.0157936)
Supplement: S1 Appendix — (DOCX) [file pone.0157936.s001.docx]

**S1 Appendix. Selected items for the eight target categories**

|  | **Birds** | **Fruit** | **Kitchen utensils** | **Mammals** | **Musical instruments** | **Tools** | **Vegetables** | **Vehicles** |
| --- | --- | --- | --- | --- | --- | --- | --- | --- |
| **Typical** | **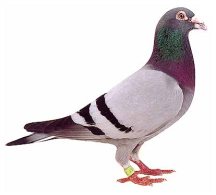** | **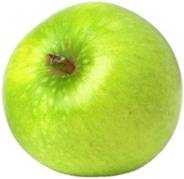** | **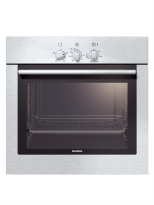** | **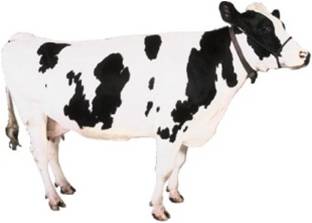** | **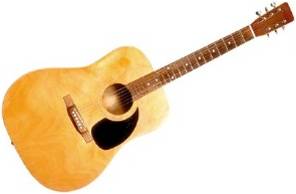** | **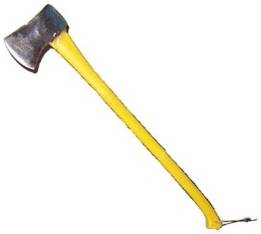** | **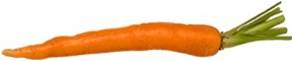** | **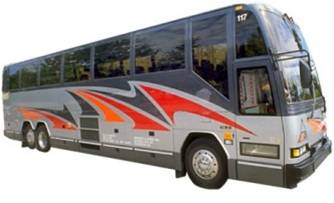** |
|  | **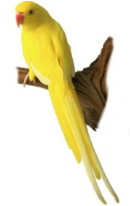** | **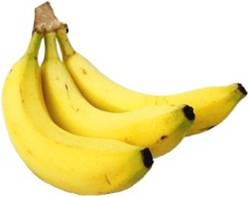** | **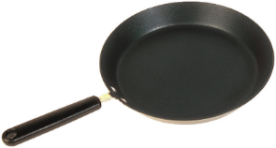** | **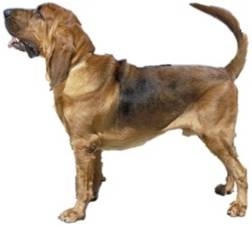** | **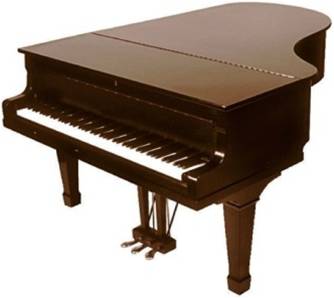** | **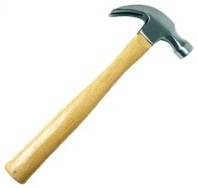** | **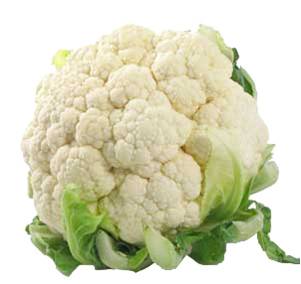** | **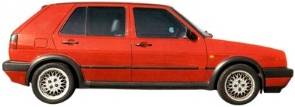** |
|  | **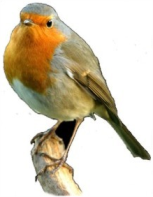** | **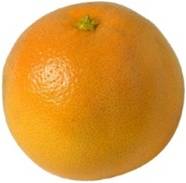** | **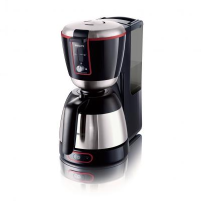** | **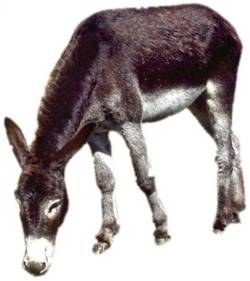** | **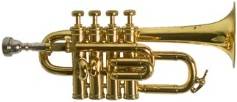** | **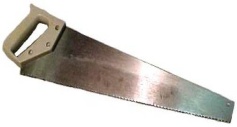** | **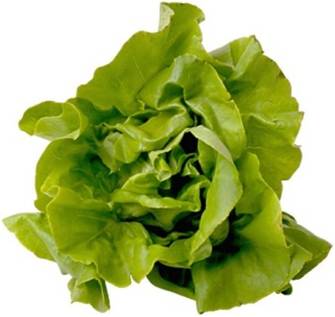** | **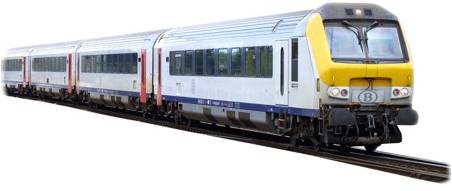** |
|  | **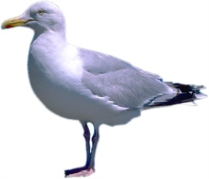** | **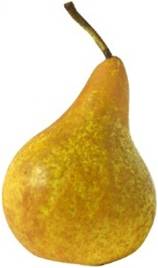** | **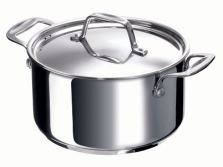** | **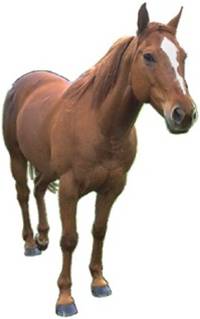** | **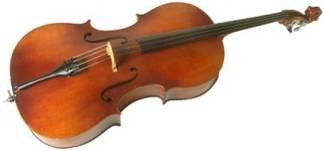** | **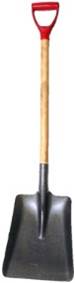** | **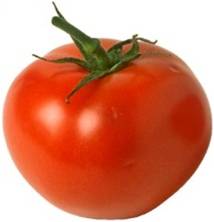** | **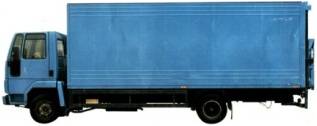** |
| **Moderate** | **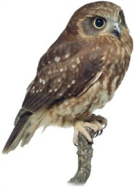** | **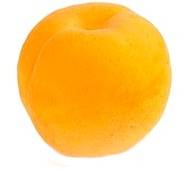** | **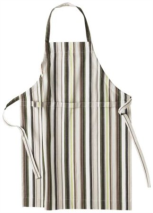** | **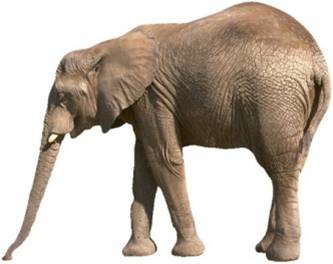** | **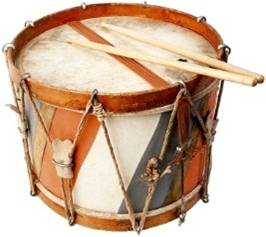** | **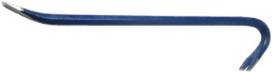** | **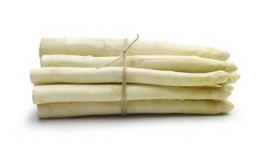** | **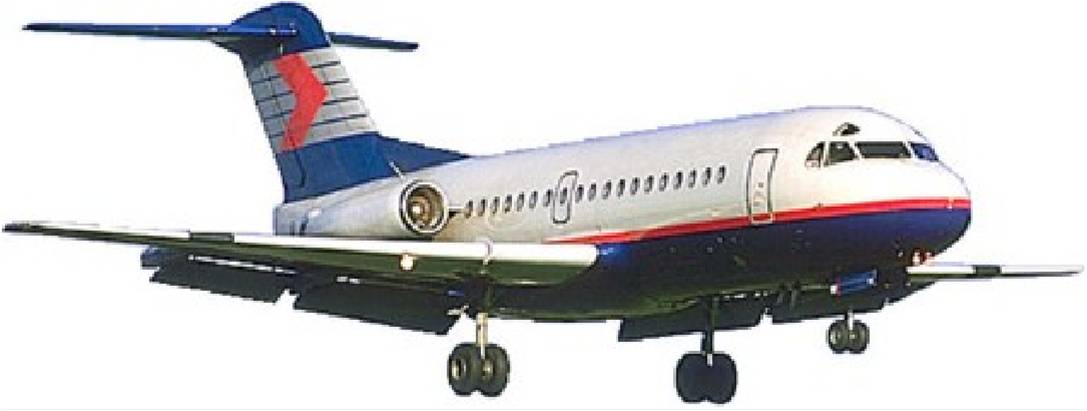** |
|  | **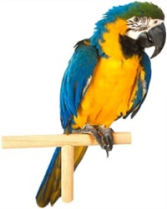** | **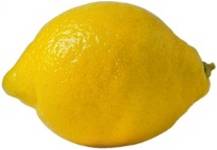** | **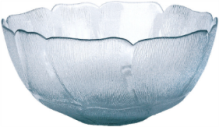** | **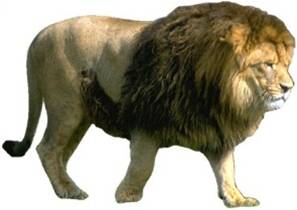** | **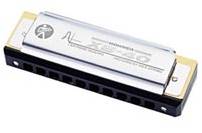** | **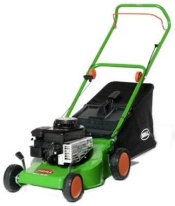** | **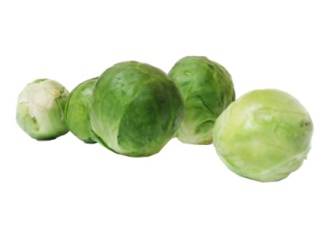** | **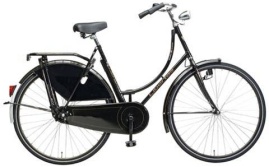** |
|  | **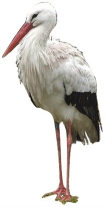** | **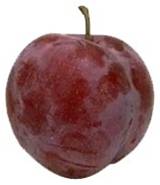** | **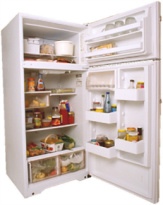** | **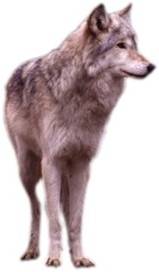** | **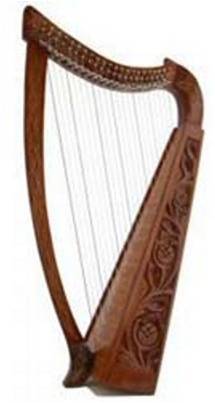** | **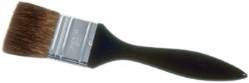** | **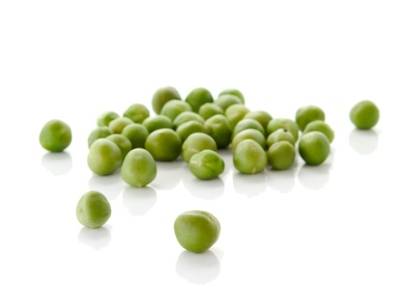** | **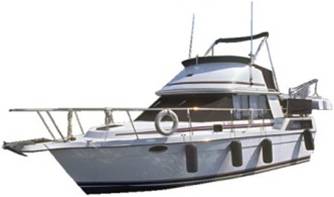** |
|  | **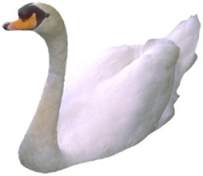** | **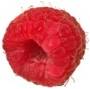** | **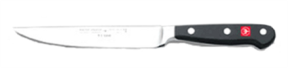** | **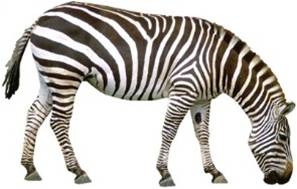** | **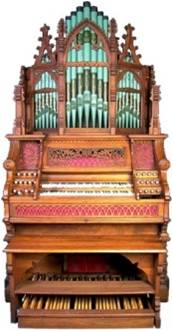** | **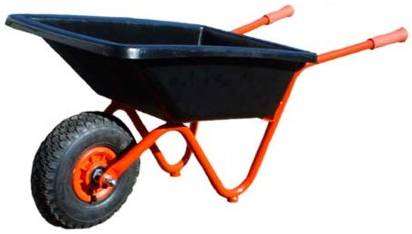** | **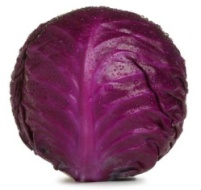** | **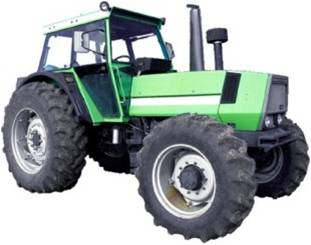** |
| **Atypical** | **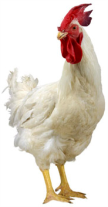** | **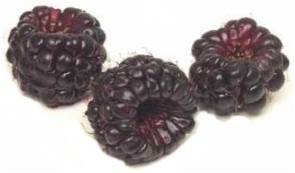** | **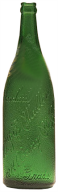** | **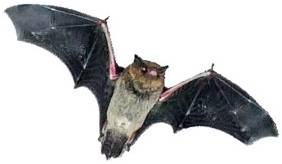** | **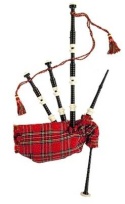** | **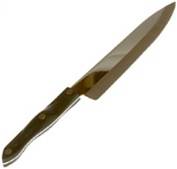** | **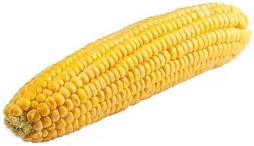** | **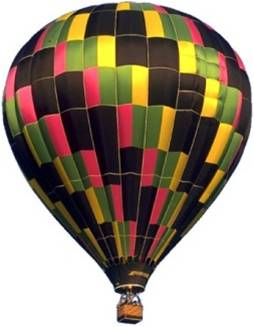** |
|  | **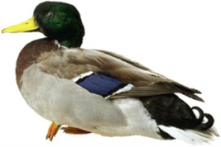** | **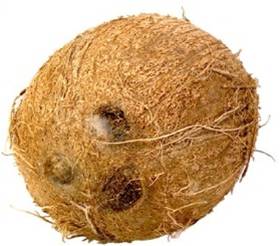** | **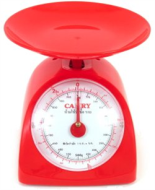** | **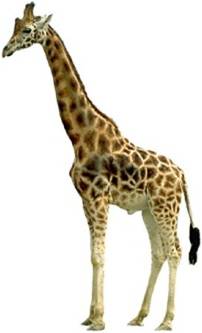** | **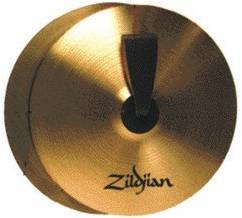** | **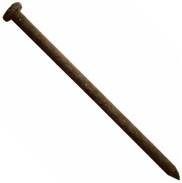** | **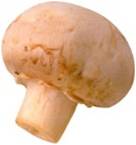** | **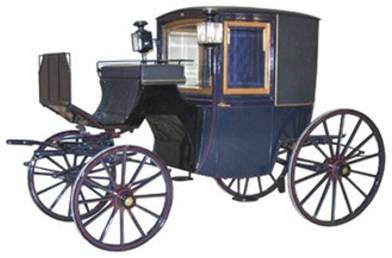** |
|  | **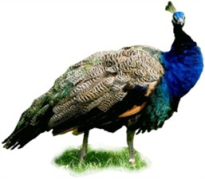** | **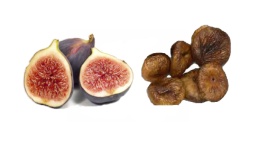** | **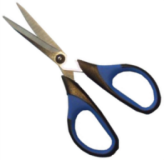** | **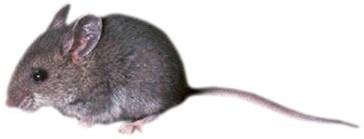** | **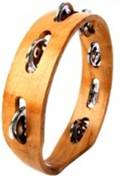** | **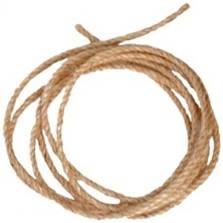** | **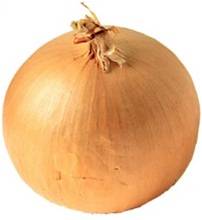** | **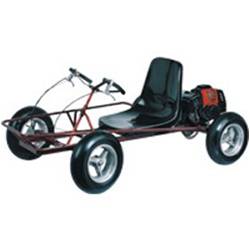** |
|  | **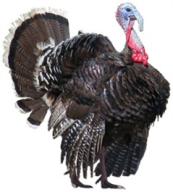** | **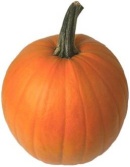** | **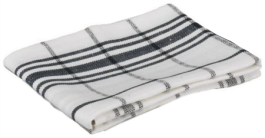** | **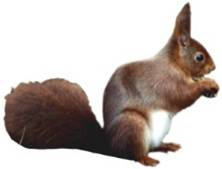** | **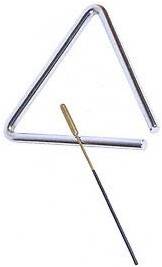** | **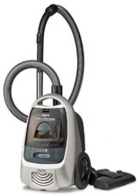** | **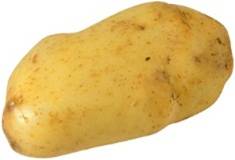** | **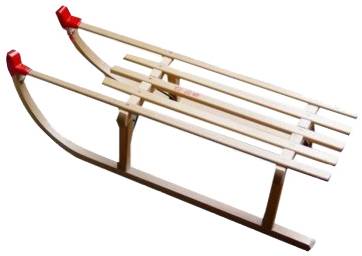** |
